# Myocardial fibrosis by CMR LGE in a large cohrt of pediatric thalassemia major patients

**DOI:** 10.1186/1532-429X-16-S1-P395

**Published:** 2014-01-16

**Authors:** Antonella Meloni, Maddalena Casale, Aldo Filosa, Blandina Pagano, Vincenzo Positano, Antonino Vallone, Gianluca Valeri, Daniele De Marchi, Massimo Lombardi, Alessia Pepe

**Affiliations:** 1 CMR Unit, Fondazione G.Monasterio CNR-Regione Toscana and Institute of Clinical Physiology, Pisa, Italy; 2 Centro per le Microcitemie, AORN Cardarelli, Napoli, Italy; 3 Centro Microcitemico, U.O. di Pediatria e Neonatologia. Presidio Ospedaliero Locri - A.S.L. n. 9, Locri, Italy; 4 Istituto di Radiologia, Az. Osp. "Garibaldi" Presidio Ospedaliero Nesima, Catania, Italy; 5 Dipartimento di Radiologia, Azienda Ospedaliero-Universitaria Ospedali Riuniti "Umberto I-Lancisi-Salesi", Ancona, Italy

## Background

Cardiovascular Magnetic Resonance (CMR) by late gadolinium enhancement (LGE) allows to detect myocardial fibrosis. Myocardial fibrosis was shown to be a relative common finding in large cohort of Italian thalassemia major (TM) patients mainly related to HCV infection, but specific studies involving only pediatric patients are not available. Our aim was to investigate the prevalence and clinical-instrumental correlates of myocardial fibrosis in pediatric TM patients.

## Methods

We studied retrospectively 76 pediatric patients with TM (44 boys, 4.2 -17.9 years old, mean age 13.6 ± 3.4 years) enrolled in the MIOT (Myocardial Iron Overload in Thalassemia) Network. All patients were well transfused and chelated since the early childhood.LGE images were acquired to detect myocardial fibrosis. Myocardial iron overload (MIO) was measured by T2* multislice multiecho technique. Biventricular function parameters were quantitatively evaluated by cine images.

## Results

Myocardial fibrosis was detected in 12 (15.8%) patients. In all patients the location of the fibrosis was epi-mesocardial, with no ischemic pattern. The youngest patient showing myocardial fibrosis had 13 years of age. Table 1 shows the comparison between patients with and without myocardial fibrosis. A significant higher MIO was detected in patients with myocardial fibrosis. The left atrial area, all the left ventricular (LV) indexed volumes, the LV mass index and the bi-ventricular stroke volume indexes were significantly higher in the fibrosis group than in the no-fibrosis group.

**Table 1:** Clinical and instrumental correlates in the fibrosis and no-fibrosis group.

	Fibrosis group (N = 12)	No-fibrosis group (N = 64)	P-value
*Sex (M/F)*	10/2	34/30	0.062

*Age (years)*	15.4 ± 1.8	13.3 ± 3.5	0.073

*Transfusions starting age (years)*	1.2 ± 0.9	1.3 ± 0.8	0.691

*Chelation starting age (years)*	3.1 ± 1.8	3.1 ± 2.3	0.705

*HCV antibodies, N (%)*	0	3 (4.8%)	0.437

*Hb pre-transfusion (g/dl)*	9.7 ± 0.3	9.5 ± 0.7	0.757

*Ferritin levels (ng/l)*	3012 ± 2167	2225 ± 1396	0.226

*ALT (u/l)*	41.6 ± 12.5	38.6 ± 32.6	0.268

*AST (u/l)*	46.6 ± 41.2	33.4 ± 25.9	0.207

Global Heart T2* (ms)	20.9 ± 13.9	30.6 ± 9.7	0.022

*MRI CIC (mg/g dry weight)*	2.0 ± 1.7	0.8 ± 0.6	0.022

Patients with global heart T2* < 20 ms, N (%)	7 (58.3)	12 (18.8)	0.008

N. of seg. with abnormal T2*	9.0 ± 7.0	3.8 ± 5.2	0.030

*Left atrial area (cm2)*	18.3 ± 3.1	15.9 ± 3.9	0.050

*Right atrial area (cm2)*	16.9 ± 4.3	14.9 ± 3.5	0.169

*Left ventricular end-diastolic volume index (ml/m2)*	102.9 ± 23.5	87.0 ± 16.3	0.005

*Left ventricular end-systolic volume index (ml/m2)*	42.0 ± 12.1	35.1 ± 8.9	0.022

*Left ventricular stroke volume index (ml/m2)*	60.7 ± 12.4	51.8 ± 10.7	0.012

*Left ventricular mass index (g/m2)*	65.3 ± 11.4	53.8 ± 11.4	0.003

*Left ventricular ejection fraction (%)*	59.2 ± 4.4	59.7 ± 5.9	0.368

*Right ventricular end-diastolic volume index (ml/m2)*	96.9 ± 25.6	81.6 ± 17.1	0.089

*Right ventricular end-systolic volume index (ml/m2)*	36.9 ± 13.7	32.3 ± 8.3	0.458

*Right ventricular stroke volume index (ml/m2)*	61.5 ± 11.6	48.9 ± 14.1	0.005

*Right ventricular ejection fraction (%)*	62.6 ± 4.4	60.2 ± 7.1	0.175

## Conclusions

In pediatric TM patients myocardial fibrosis is not a rare finding to keep in mind in the cardiological management. When appropriate treatment has been administered since early childhood, CMR LGE can be postponed until 13 years of age. By the natural history of this large cohort of pediatric patients where HCV infection has been appropriately prevented, myocardial fibrosis seem to be associated with MIO and high cardiac output.

## Funding

The MIOT project receives "no-profit support" from industrial sponsorships (Chiesi Farmaceutici S.p.A. and ApoPharma Inc.). This study was also supported by: "Ministero della Salute, fondi ex art. 12 D.Lgs. 502/92 e s.m.i., ricerca sanitaria finalizzata anno 2006" and "Fondazione L. Giambrone".

